# A New Stability Indicating HPLC Method for Related Substances in Zolmitriptan

**DOI:** 10.4103/0250-474X.62252

**Published:** 2010

**Authors:** E. K. S. Vijayakumar, M. A. Samel, S. B. Bhalekar, S. M. Pakhale

**Affiliations:** Mylan India Pvt. Ltd., Plot 1A/2, M.I.D.C. Industrial Estate, Taloja, Panvel-410 208, India

**Keywords:** Forced degradation, HPLC, method development, related substances, zolmitriptan

## Abstract

A sensitive, precise, specific, linear and stability indicating isocratic HPLC method was developed for the analysis of related substances in zolmitriptan. The potential known related substances are (S)-4-(4-aminobenzyl)-1,3-oxazolidin-2-one (impurity I) and (S)-4-(4-hydrazinobenzyl)-1,3-oxazolidin-2-one (impurity II). The method can be used for the detection and quantification of known and unknown impurities and degradants in the drug substance zolmitriptan during routine analysis and also for stability studies in view of its capability to separate degradation products.

Zolmitriptan is a synthetic triptamine derivative and is chemically known as (S)-4-{[3-(2-dimethylaminoethyl)-1H-indol-5-yl]methyl}-1,3-oxazolidin-2-one. Zolmitriptan is an oral, selective serotonin receptor agonist of the serotonin receptor 1B/1D (5-HT_1B_/_1D_) and is used for the treatment of acute migraine attacks[1–3]. It causes constriction of the blood vessels thereby relieving the pain due to migraine headache.

Several analytical HPLC methods were reported in literature for the quantitative determination of zolmitriptan and its metabolites in human plasma and other biological fluids[4–11]. Very few achiral and chiral HPLC methods were reported for the detection and quantification of related impurities in the drug substance zolmitriptan[12–14].

Zolmitriptan was synthesized as per the synthesis route described in a patent[15]. The known related substances, which may be present in the bulk samples of zolmitriptan are the precursors (S)-4-(aminobenzyl)-1,3-oxazolidin-2-one (impurity I) and (S)-4-(4-hydrazinobenzyl)-1,3-oxazolidin-2-one (impurity II, fig. 1). Evaluation of the reported achiral HPLC methods were found to be unsuitable for the simultaneous detection and quantification of impurities I and II in the drug substance zolmitriptan due to unsatisfactory peak shapes, thus, necessitating us to develop a new HPLC method for the detection and quantification of impurities I and II in zolmitriptan.

**Fig. 1:**
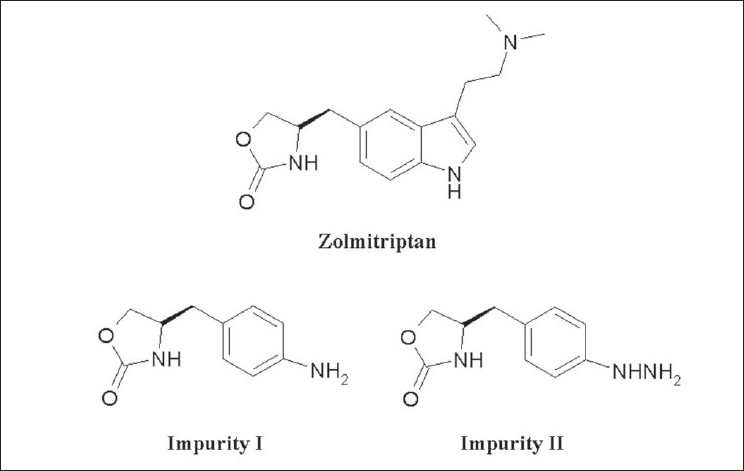
Chemical structures of zolmitriptan and impurities I and II Zolmitriptan: (S)-4-{[3-(2-dimethylaminoethyl)-1H-indol-5-yl] methyl}-1,3-oxazolidin-2-one; Impurity I: (S)-4-(aminobenzyl)-1,3-oxazolidin-2-one; Impurity II: (S)-4-(4-hydrazinobenzyl)-1,3-oxazolidin-2-one.

In this paper, we report the development of a new isocratic stability indicating HPLC method for the simultaneous detection and quantitative determination of the impurities I and II in zolmitriptan. Forced degradation studies[16] were carried out to establish stability indicating nature of the method. System suitability, limit of detection (LOD), limit of quantification (LOQ) and linearity were established as per ICH Guidelines[17,18].

Samples of zolmitriptan and the impurities I and II were synthesized and characterized at Mylan India Pvt. Ltd. (formerly Merck Development Centre, India). HPLC grade acetonitrile was procured from Merck Specialities Pvt. Ltd. and analytical grade ammonium formate was procured from Sigma Aldrich. High purity water was generated in-house from Elga option-R7 water purification system. HPLC system used for this study was Waters Alliance HPLC system using Waters Empower Net Working Software. A photo diode array detector (Model No. 2996) was used during forced degradation studies, while LOD, LOQ and Linearity data was obtained using a UV detector (Model No. 2487).

The mobile phase consisted of a mixture of 0.02 M ammonium formate containing 0.1% *n*-propylamine and acetonitrile in 80:20 v/v, which was filtered through a 0.45 μ nylon membrane and degassed by sonication before use. The chromatographic column used was Waters XTerra C18 (250*4.6 mm), 5 μ. The chromatography was performed at a flow rate of 1.0 ml/min using the above mentioned mobile phase. The column temperature was maintained at 33° and the detection wavelength was 225 nm.

A solution of a mixture of 0.5 mg/ml (0.05% w/v) of zolmitriptan and 750 ng/ml each of the impurities I and II was prepared in diluent (50:50 v/v mixture of water-acetonitrile) for method development and system suitability. Stock solutions of 0.1 mg/ml (0.01% w/v) of zolmitriptan and the impurities I and II were prepared separately in diluent. From these stock solutions, six different solutions of mixtures of zolmitriptan and the impurities I and II were prepared in the range of 10 ng/ml to 1000 ng/ml and analyzed by using the above HPLC method. LODs were determined from visual observation of areas of zolmitriptan and impurities I-II at each concentration in comparison with background obtained by injecting a blank. LOQs were considered as 3 times of LODs. After determining LOD and LOQ for zolmitriptan and impurities I-II, two separate mixtures of zolmitriptan and impurities I and II in diluent having concentrations equivalent to LODs and LOQs respectively were prepared and six replicate injections of each of these solutions were performed to establish precision at LOD and LOQ level.

Six solutions containing known concentrations of zolmitriptan and the impurities I and II were prepared at different concentration levels in the range of 150 ng/ml to 1000 ng/ml for zolmitriptan, 150 ng/ml to 1500 ng/ml for impurity I and 600 ng/ml to 1500 ng/ml for impurity II from the above stock solutions. Stock solutions of zolmitriptan (I) containing 10 mg/ml each were prepared in aqueous 1.0 N HCl, 0.1 N NaOH and 0.1 N H_2_O_2_. Additionally, 0.5 mg/ml (0.05% w/v) solution of zolmitriptan was prepared in diluent as a control sample. All the solutions were kept at ambient temperature for 1 to 5 days. For HPLC analysis, 2.5 ml each of stock solutions were diluted to 50 ml with diluent to get 0.5 mg/ml (0.05% w/v) solutions.

Based on the absorption maxima observed for all the three components, the detection wavelength was set at 225 nm. Different octadecylsilyl silica columns like Lichrospher RP 18e, Waters Symmetry, Inertsil ODS 3V, Hypersil ODS and Waters XTerra were used during method development. Mobile phases comprising of phosphate, acetate or formate buffer in combination with methanol or acetonitrile or both were tried under gradient and isocratic conditions to obtain separation between the components. In most cases, zolmitriptan was not retained on the column, besides unsatisfactory peak shape for impurity II and co-elution of an unknown impurity with zolmitriptan. The effects of pH of mobile phase and column oven temperature on resolution between the components and tailing factors were also studied. A reasonably good separation between the known and unknown impurities from zolmitriptan with good peaks shapes was achieved with 0.02 M ammonium formate containing 0.1% *n*-propylamine and acetonitrile in (80:20 v/v) on Waters XTerra (250*4.6 mm, 5 μ) at a flow rate of 1.0 ml/min and column oven temperature of 33°. The retention times of zolmitriptan, impurities I and II are around 11.0, 4.7 and 27.6 min, respectively with a resolution of 13.5 between zolmitriptan and impurity I and 24.1 between zolmitriptan and impurity II. Tailing factor for all the three components was 1.0.

LOD and LOQ were determined to be 50 ng/ml (% RSD= 2.19) and 150 ng/ml (% RSD= 1.21), respectively for zolmitriptan. LOD and LOQ were found to be 50 ng/ml (% RSD = 2.09) and 150 ng/ml (% RSD= 2.16), respectively for impurity I. LOD and LOQ were 200 ng/ml (% RSD= 3.06) and 600 ng/ml (% RSD= 0.72), respectively for impurity II. The linearity was established in the range of 150 ng/ml to 1000 ng/ml for zolmitriptan, 150 ng/ml to 1500 ng/ml (LOQ level to 200% of the specified limit of 0.15% in zolmitriptan) for impurity I and 600 ng/ml to 1500 ng/ml (LOQ level to 200% of the specified limit of 0.15% in zolmitriptan) for impurity II. The regression coefficients were 0.9995, 0.9998 and 0.9952 respectively. Representative chromatograms of a mixture of zolmitriptan, impurities I and II at LOD level and also at LOQ level are shown in figs. 2 and 3. Analysis of zolmitriptan samples using the above method indicated the presence of unknown impurities in the range of 0.03-0.07% by area normalization. A typical chromatogram of a zolmitriptan sample indicating the detection and separation of unknown impurities is shown in fig. 4. This indicated that the method was sensitive and capable of detecting and quantifying known impurities I and II and unknown impurities as well.

**Fig. 2:**
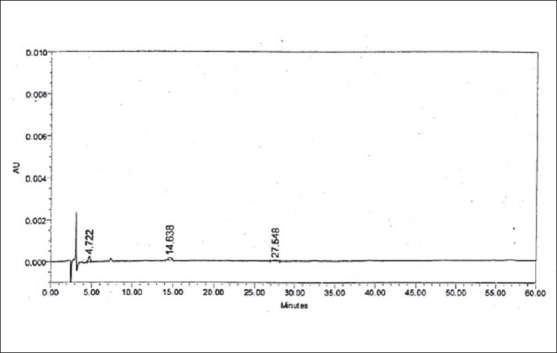
Chromatogram of a mixture of zolmitriptan and impurities I-II at LOD level Peak at t_R_: 4.722 min is due to impurity I (LOD: 50 ng/ml); Peak at t_R_: 14.638 min is due to zolmitriptan (LOD: 50 ng/ml); Peak t_R_: 27.548 min is due to impurity II (LOD: 200 ng/ml).

**Fig. 3:**
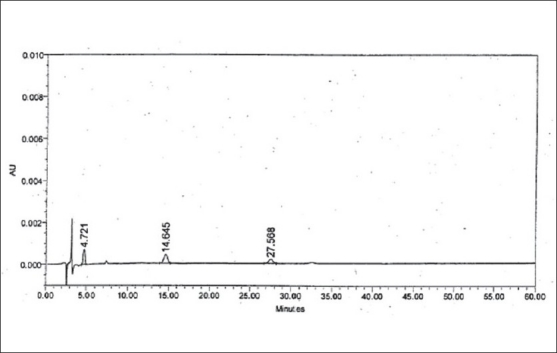
Chromatogram of a mixture of zolmitriptan and impurities I-II at LOQ level Peak at t_R_: 4.721 min is due to impurity I (LOQ: 150 ng/ml); Peak at t_R_: 14.645 min is due to zolmitriptan (LOQ: 150 ng/ml); Peak t_R_: 27.568 min is due to impurity II (LOQ: 600 ng/ml).

**Fig. 4:**
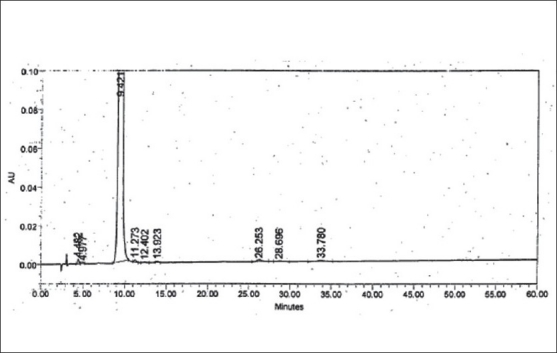
Chromatogram of a zolmitriptan sample Peak at t_R_: 4.482 min is due to impurity I; Peaks at t_R_: 4.977, 11.273, 12.402, 13.923, 26.253, 28.696 and 33.780 min are due to unknown impurities; Peak at t_R_: 9.421 min is due to zolmitriptan.

Table 1, summarizes the results obtained from forced degradation studies. No degradation was observed for zolmitriptan in diluent and under acidic conditions after 5 days. Zolmitriptan degraded by about 15.25% in 4 days under alkaline conditions. About 15.2% by area normalization of a major degradant was observed at RRT 0.51, which could be attributed to (2S)-2-amino-3-[3-(2-dimethyl aminoethyl)-1H-5-indolyl]-propan-1-ol[14]. Under oxidative conditions, degradation was observed for zolmitriptan by about 9.69% after 1 day. Two unknown degradants (RRT 0.28: 7.4%, RRT 0.35: 1.9% by area normalization) were observed. The peak purity analysis of all the samples using PDA detector confirmed that there were no co-eluting peaks in the principal peak of zolmitriptan, thus, establishing the stability indicating nature of the method.

**TABLE 1:** SUMMARY OF FORCED DEGRADATION STUDIES FOR ZOLMITRIPTAN

Stress condition	% Purity	% Degradation	Degradation products
Control (Initial, 0 h)	99.42	-	-
Diluent (ambient, 5 days)	99.42	No degradation	-
1 N HCl (ambient, 5 days)	99.45	No degradation	-
0.1 N NaOH (ambient, 5 days)	84.17	15.25	15.2% (RRT: 0.51)
0.1 N H_2_O_2_ (ambient, 1 day)	89.73	9.69	7.4% (RRT: 0.28)
			1.9% (RRT: 0.35)

A new isocratic HPLC method has been developed for the analysis of related substances in zolmitriptan. LOD, LOQ and Linearity were determined for zolmitriptan and the impurities I-II. The method was established to be specific and stability indicating. It is simple, sensitive, linear and precise for the simultaneous detection and quantification of known and unknown impurities and degradants in zolmitriptan. The method can be used for the routine and stability analysis of drug substance and dosage forms.

## References

[1] Oldman AD, Smith LA, McQuay HJ, Moore RA (2002). A systematic review of treatments for acute migraine. Pain.

[2] Yates R, Nairn K, Dixon R, Seaber E (2002). Preliminary studies of the pharmacokinetics and tolerability of zolmitriptan nasal spray in healthy volunteers. J Clin Pharmacol.

[3] Yates R, Nairn K, Dixon R, Kemp JV, Dane AL (2002). Pharmacokinetics, dose proportionality and tolerability of single and repeat doses of a nasal spray formulation of zolmitriptan in healthy volunteers. J Clin Pharmacol.

[4] Chen X, Liu D, Luan Y, Jin F, Zhong D (2006). Determination of zolmitriptan in human plasma by liquid chromatography-tandem mass spectrometry method: Application to a pharmacokinetic study. J Chromatogr B.

[5] Yu L, Wen Y, Song Z, Mu D, Su L, Yang Y (2006). Determination of zolmitriptan and its pharmacokinetics in human plasma after intranasal administration using LC-MS. Fenxi Ceshi Xuebao.

[6] He H, Meng H, Zhou Y, Li B, Li X (2005). Determination of zolmitriptan in human plasma by RP-HPLC with liquid-liquid extraction. Yaowu Fenxi Zazhi.

[7] Yao J, Qu Y, Zhao X, Hu L, Zhu R, Li H (2005). Determination of zolmitriptan in human plasma by high performance liquid chromatography-electrospray mass spectrometry and study on its pharmacokinetics. J Chinese Pharm Sci.

[8] Zang Z, Xu F, Tian Y, Li W, Mao G (2004). Quantification of zolmitriptan in plasma by high-performance liquid chromatography-electrospray ionization mass spectrometry. J Chromatogr B Analyt Technol Biomed Life Sci.

[9] Chen J, Jiang X, Jiang W, Mei N, Gao X, Zhang Q (2004). High performance liquid chromatographic analysis of zolmitriptan in human plasma using fluorescence detection. J Pharm Biomed Anal.

[10] Clement EM, Franklin M (2002). Simultaneous measurement of zolmitriptan and its major metabolites N-desmethylzolmitriptan and zolmitriptan N-oxide in human plasma by high-performance liquid chromatography with coulometric detection. J Chromatogr B Analyt Technol Biomed Life Sci.

[11] Kilic B, Özden T, Toptan S, Özilhan S (2007). Simultaneous LC-MS-MS determination of zolmitriptan and its active metabolite N-desmethylzolmitriptan in human plasma. Chromatographia.

[12] Hu Y, Yao T, Wang X (2004). HPLC determination of zolmitriptan and its related substances. Zhejiang Daxue Xuebao, Yixueban.

[13] Srinivasu MK, Mallikarjuna Rao B, Sridhar G, Rajender Kumar P, Chandra Sekhar KB, Aminul I (2005). A validated chiral LC method for the determination of zolmitriptan and its potential impurities. J Pharm Biomed Anal.

[14] Mallikarjuna Rao B, Srinivasu MK, Sridhar G, Rajender Kumar P, Chandra Sekhar KB, Aminul I (2005). A stability indicating LC method for zolmitriptan. J Pharm Biomed Anal.

[15] Gore VG, Datta D, Gadkar MS, Pokharkar KS, Phatangare K A convenient process (Zolmitriptan).

[16] Q1A (R2) (2003). ICH Harmonized Tripartite Guideline.

[17] Q2A (1994). ICH Harmonized Tripartite Guideline.

[18] Q2B (1996). ICH Harmonized Tripartite Guideline.

